# Improved Facial Nerve Identification with Novel Fluorescently Labeled Probe

**DOI:** 10.1002/lary.21411

**Published:** 2011-02-16

**Authors:** Amy P Wu, Michael A Whitney, Jessica L Crisp, Beth Friedman, Roger Y Tsien, Quyen T Nguyen

**Affiliations:** Division of Otolaryngology—Head and Neck Surgery, UCSDSan Diego, California, U.S.A.; Department of Pharmacology, UCSDSan Diego, California, U.S.A.; Department of Chemistry and Biochemistry, UCSDSan Diego, California, U.S.A.; Howard Hughes Medical Institute, Departments of Pharmacology, Chemistry and Biochemistry, UCSDSan Diego, California, U.S.A.

**Keywords:** Facial nerve injury, anastomosis, fluorescent microscopy, nerve labeling, Level of Evidence: N/A

## Abstract

**Objectives/Hypothesis:**

By phage display, we have developed a novel peptide (NP41) that binds selectively to nerves following systemic administration. We evaluated the pattern of facial nerve labeling with fluorescently-labeled NP41 (F-NP41). We also tested whether F-NP41 highlights facial nerves well enough to identify nerve stumps accurately several weeks after nerve transection.

**Study Design:**

Forty-seven wild-type mice were studied prospectively. One surgeon performed the nerve transection, reanastomoses, and monitoring of functional recovery.

**Methods:**

Fluorescent labeling: F-NP41 was administered intravenously (20 mice). Nerve labeling was studied with fluorescence microscopy. Transection and reanastomosis: the right facial nerve was transected (25 mice). Three weeks after transection, F-NP41 was administered intravenously and fluorescence microscopy was used to identify the nerve stumps and reanastomosis in one group. Nerve identification and renastomosis was performed with white light in another group without F-NP41. The control group underwent sham surgery. Time to nerve identification was recorded. Functional recovery was monitored for at least 8 weeks.

**Results:**

We found excellent labeling of intact and transected facial nerves following F-NP41 administration. Several weeks following nerve transection, F-NP41 provided accurate identification of the proximal and distal nerve stumps. Following reanastomosis, time to recovery and level of functional recovery was similar in the absence and presence of F-NP41.

**Conclusions:**

We show improved visualization of facial nerves with a novel systemically applied fluorescently labeled probe. Use of F-NP41 resulted in accurate identification of facial nerve stumps several weeks following transection. Functional recovery was similar with and without the use of F-NP41. Laryngoscope, 2011

## INTRODUCTION

Surgical identification of traumatically or iatrogenically transected facial nerve can be challenging due to degeneration of distal branches. Currently facial nerve dissection and anastomosis are recommended for facial nerve injuries within 3 days of trauma.^1^, ^2^ During this time, the distal nerve branches may be easily located because they can still be stimulated electrically.^1^ Several months to a year after trauma, electromyographic tracing is no longer possible, yet if the nerves can be found, repair or grafting still results in a good functional outcome.^3^, ^4^

We have previously described a novel probe that binds nerves following systemic administration.^5^ In this study, we tested whether or not the novel fluorescently labeled probe (F-NP41) highlights facial nerves before and after transection. We also tested whether the use of F-NP41 improved the accuracy of nerve stump identification after transection and functional recovery after reanastomosis.

## MATERIALS AND METHODS

### Fluorescently labeled Nerve Probe

**Fig. 1:**
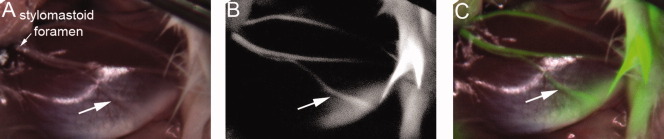
Right lateral face of a wild-type mouse treated with F-NP41. (A) White-light image of the right facial nerve. (B) Fluorescence image of the F-NP41-labeled facial nerve. (C) Composite image superimposing the fluorescence image with the white-light image. The arrows show a small nerve branch on the masseter muscle that is not clearly observed in white light (A), but becomes apparent with fluorescence (B,C). [Color figure can be viewed in the online issue, which is available at wileyonlinelibrary.com.]

**Fig. 2:**
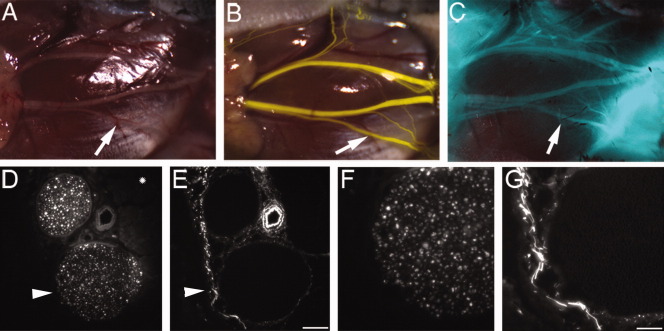
Right lateral face of a thy1-YFP transgenic mouse treated with Cy5-NP41. A. White-light image of the right facial nerve. (B) Fluorescence image of axonal YFP in the same mouse as A. (C) Fluorescence image of Cy5-NP41 in the same mouse as A and B. The arrows show a small nerve branch on the masseter muscle that is not clearly observed in white light (A), but becomes apparent with fluorescence (B, C). (D) Fluorescence microscopy of a 3-μm-thick cross-section of the right facial nerve of a thy1-YFP transgenic mouse treated with Cy5-NP41. The arrowhead points to the nerve bundle with adjacent muscle fibers (*). YFP is seen in axoplasm. (E) In the same section as (D), Cy5-NP41 uptake is seen in epineurium (scale bar = 50 μm). (F–G) Higher magnification of same sections in D and E (scale bar = 25 μm). [Color figure can be viewed in the online issue, which is available at wileyonlinelibrary.com.]

F-NP41 was synthesized as previously described.^5^ For the visualization of facial nerves before and after transection, the fluorescent dye was carboxyfluorescein conjugated to a C-terminal lysinamide, resulting in Ac- SHSNTQTLAKAPEHTGK(5,6FAM)-amide (AnaSpec, Fremont, CA) (Fig. 1). For the visualization of facial nerves in transgenic animals expressing axonal Yellow Fluorescent Protein (YFP), fluorescein would have excessive spectral overlap with YFP, so the fluorescent label was switched to Cy5-maleimide (GE Healthcare, UK) attached to a C-terminal cysteinamide (Fig. 2).

### Transgenic Animals with Axonal YFP Expression

Transgenic mice expression of YFP under a neuron specific promoter^6^ (strain name B6.Cg-Tg[Thy1-YFP]16Jrs/J, stock number 003709) were obtained from Jackson Laboratories (Bar Harbor, ME) and bred in our animal facility. These mice express YFP at high levels in motor and sensory neurons.

### Fluorescent Labeling of Facial Nerves

Following anesthesia with intraperitoneal injection of ketamine (50 mg/kg) and midazolam (1 mg/kg), 150 nmol of F-NP41 was administered intravenously via tail vein injection into wild-type mice. Following a washout period of 2 to 3 hours for carboxyfluorescein-labeled NP41 and 6 to 7 hours for Cy5-labeled NP41, facial nerves were imaged using a Zeiss Lumar fluorescent dissecting microscope or a custom-made surgical fluorescence imaging system based on an Olympus dissecting microscope. Carboxyfluorescein and YFP were imaged with 450–490 nm excitation and 500–550 nm emission. Cy5 was imaged with 590–650 nm excitation and 663–738 nm emission.

### Facial Nerve Transections

**Fig. 3:**
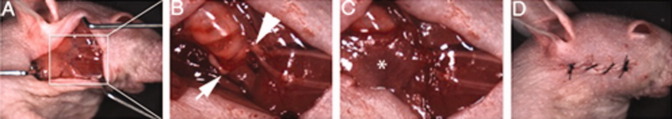
Steps of right facial nerve transection in a wild-type SKH1 mouse. (A) After an infraauricular skin incision, the parotid gland is seen overlying the proximal peripheral facial nerve. (B) At higher magnification, the parotid gland has been partially resected and retracted, and the main trunk of the facial nerve has been cut and released from the surrounding fascia. The arrow highlights the proximal stump. The arrowhead points to the distal stump. (C) The remaining portion of the parotid gland (*) is reflected back down. (D) The skin is closed in a single layer. [Color figure can be viewed in the online issue, which is available at wileyonlinelibrary.com.]

Twenty-five adult, wild-type albino SKH1 mice (Charles River, Wilmingham, MA), weighing 23 to 28 g, were used as our nerve transection model. Briefly, each animal was anesthetized with intraperitoneal ketamine (50 mg/kg) and midazolam (1 mg/kg). A 1.5-cm lateral skin incision was made inferior to the external auditory canal (EAC) along the course of the facial nerve (Fig. 3A). The anterior aspect of the parotid gland was dissected off the underlying branches of the facial nerve and reflected posteriorly. The external jugular vein and its branches lying superficial to the pes anserinus were heat-cauterized as needed. The main trunk of the facial nerve was followed as it wrapped around the inferior border of the EAC and anterior to the posterior belly of the digastric muscle. The nerve was atraumatically released from the surrounding tissues with meticulous dissection under microscopic visualization. A sharp cut was made through the main trunk with microscissors. The proximal (Fig. 3B, arrow) and distal (Fig. 3B, arrowhead) cut ends were meticulously released from connective tissue attachments to adjacent structures to allow retraction from each other and to ensure no spontaneous recovery. The parotid gland was reflected back down to provide a layer of protection between the nerve and the overlying incision (Fig. 3C). The wound was closed in a single layer (Fig. 3D). Complete nerve transection was verified by the lack of ipsilateral whisker movement or alar flaring under microscopic magnification.^7^ All animal studies were approved by the UCSD Institutional Animal Care and Use Committee (Protocol number S05536).

**Fig. 4:**
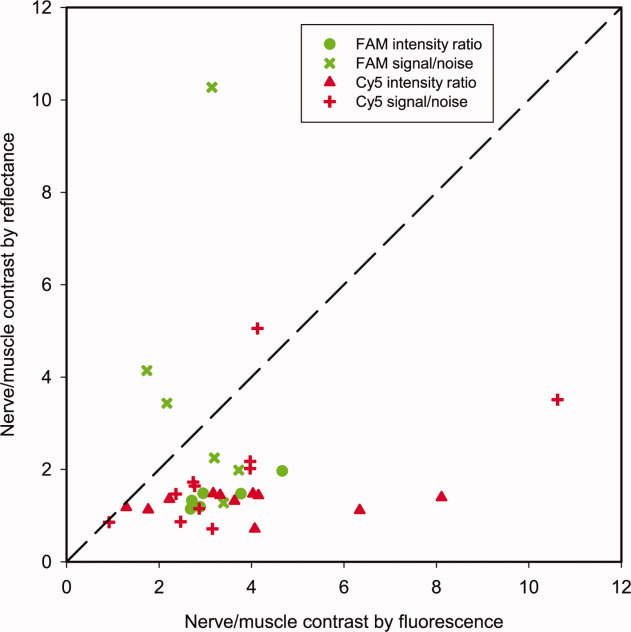
Intensity and signal to noise ratios for reflectance and fluorescence measurements were plotted for individual nerve branches. Equal performance of reflectance versus fluorescence is indicated by dashed line (slope = 1). Values to the right of the line indicate that there is improved visualization with fluorescence images and values to the left of the line indicate that there is improved visualization with reflectance images. [Color figure can be viewed in the online issue, which is available at wileyonlinelibrary.com.]

### Analysis of Simple Ratio and Signal-to-Noise Ratio

Facial nerves were imaged with white light reflectance and fluorescence with the customized Olympus microscope. Gain and background subtraction were individually optimized for both fluorescence and reflectance images. Nerves and adjacent nonnerve tissue were hand-selected using arbitrary shape tool in ImageJ. The mean and standard deviation of the pixel intensities within the selected areas were compared for individual nerve branches (mean = *I*_*n*_, SD = σ_*n*_) and adjacent muscle (mean = *I*_*m*_, SD = σ_*m*_). One measure of contrast is the simple ratio of means, *I*_*n*_*/I*_*m*_. A difference signal-to-noise ratio that allows background subtraction and takes noise into account is ∥*I*_*n*_ − *I*_*m*_|/(σ_*n*_^2^ + σ_*m*_^2^)^0.5^. Results of both simple and signal-to-noise ratios for both reflectance and fluorescence were plotted for individual nerve branches (Fig. 4).

### Facial Nerve Reanastomosis

**Fig. 5:**
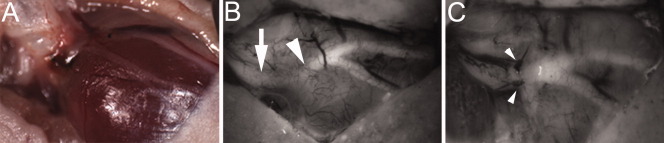
Approximately 3 weeks after initial transection, the right facial nerve is viewed under bright field and fluorescent microscopy in the group treated with F-NP41. (A) The injured stumps are not easily identifiable under bright-field view. (B) The identification of the injured facial nerve stumps in a similar mouse is aided by the use of F-NP41 under fluorescence microscopy. The formation of scar tissue and/or neuroma after injury to this region increases the difficulty of localizing the stumps. The arrow points to the approximate location of the proximal stump. The arrowhead highlights the approximate location of the distal stump. (C) After excision of scar tissue, the two stumps are anastomosed with 10-0 sutures (small arrowheads). [Color figure can be viewed in the online issue, which is available at wileyonlinelibrary.com.]

In group 1, seven mice were used as controls. They underwent facial nerve transections but not reanastomosis. Sham surgery consisting of reopening and reclosing the incision was performed 3 weeks later. In group 2, nine mice underwent facial nerve reanastomosis 3 weeks after transection without F-NP41. In group 3, nine mice underwent facial nerve reanastomosis 3 weeks after transection with the use of F-NP41 and fluorescent optical microscopy to aid in nerve stump identification. For this last group, reanastomosis was performed 2 hours after intravenous injection of F-NP41. Nerve reanastomoses in groups 2 and 3 were performed in the following manner: the animal was anesthetized with intraperitoneal ketamine and midazolam as described above. A 0.8-cm vertical incision was made below the EAC. The parotid gland was dissected off. Meticulous dissection was performed until the proximal and distal stumps of the previously transected facial nerve were identified. Two interrupted 10-0 nylon sutures (Ethicon, San Angelo, TX) were used to achieve a tension-free anastomosis. The F-NP41 labeled group was additionally studied with fluorescent optical microscopy to confirm reapproximation of the nerve ends (Fig. 5B and C). The skin incision was closed and the animals returned to their cages to recover from anesthesia.

### Monitoring of Facial Nerve Function

The control group was monitored weekly for at least 8 weeks. For the two anastomosed experimental groups, facial nerve function was monitored over at least 15 weeks. Facial nerve function was determined as any whisker movement and/or alar flaring compared to the contralateral control side. Eye blink has been found to be an unreliable measure of independent facial nerve function at least in rats.^8^ The time from nerve anastomoses to any sign of facial nerve function was recorded. Surgical loupes were used to assist with identification of facial movement. Return of function was graded none, mild, or moderate.

### Data Analysis

Pearson's chi-square test and Student's *t*-test were used to compare the groups. A significant value was considered *P ≤*.05. The mean operative time to positive identification of the proximal and distal stumps of the facial nerve were recorded and analyzed. The time from nerve reanastomoses to any sign of facial nerve function was recorded and analyzed.

## RESULTS

### Adverse Effects of F-NP41

There was no morbidity or mortality associated with F-NP41 treatment. All mice treated with F-NP41 showed similar generalized activity, grooming, feeding, and weight gain compared to control mice during the entire study period.

### Fluorescent Labeling of Facial Nerves

To study the pattern of facial nerve labeling with our nerve probe, we injected F-NP41 into wild-type mice and viewed facial nerves with fluorescent microscopy after skin excision. F-NP41 accurately labeled all branches of the facial nerve and increased contrast over that seen with white-light illumination (n = 20, Fig. 1). We also found that nerves not present on the surface of the operative field were more easily identified with the fluorescent labeling compared to white-light illumination (Fig. 1A–C, arrow).

### Facial Nerve Labeling with F-NP41 Showed a Pattern Similar to Transgenic Axonal YFP Expression

To evaluate the localization of NP41 binding in nerves, we treated thy1-YFP transgenic mice whose axons are genetically encoded with YFP under a neuron specific promoter^6^ with Cy5 labeled NP41. We found that Cy5-NP41 (Fig. 2C) precisely labeled nerves that are genetically encoded with YFP (Fig. 2B) and as seen with bright-field imaging (Fig. 2A). To evaluate the localization of NP41 binding on a cellular level, we imaged cryosections (3-μm thickness) of frozen nerves and attached muscles from thy1-YFP animals treated with Cy5-NP41 (Fig. 2D–G). We found that Cy5-NP41 appears to be most localized to the epineurium of the nerves (Fig. 2E and G) with limited labeling of the perineurium and endoneurium. We also found that NP41 labeling (Fig. 2G, high power) does not appear to colocalize with either myelin or axons. We plan a systemic evaluation of proteins that exhibit a similar pattern of localization to obtain insight into the molecular target(s) for NP41.

### Facial Nerve Branches Are Easier to Identify with F-NP41 Labeling Than with White light reflectance alone. We compared the ease of identification of facial nerve Branches in Wild-Type Mice Using Fluorescence Imaging Following F-NP41 Treatment versus White-Light Reflectance

We found that the average intensity ratio of nerve to nonnerve tissue measured from white-light reflectance images = 1.3 ± 0.26 (mean ± SD, n = 17). In contrast, the average ratio of nerve to nonnerve tissue measured from fluorescent images following F-NP41 (FAM-NP41 and Cy5-NP41) administration were 3.3 ± 0.79 (n = 6, *P* = .0003) and 3.8 ± 1.9 (n = 11, *P* = .00038) respectively. Results of both intensity ratios and signal to noise ratios between reflectance and fluorescence measurements were plotted for individual nerve branches (Fig. 4). Equal performance of the two modalities would be along a straight line with a slope of 1. Values to the left and right of the line indicate improved visualization with reflectance and fluorescence, respectively. Using both methods of analysis (simple and signal to noise ratio), the majority of the values fall to the right of the line, indicating that fluorescence imaging with FAM-NP41 or Cy5-NP41 improve visualization of nerve branches compared to white-light reflectance alone.

### Facial Nerve Stump Identification

To evaluate whether or not F-NP41 can label proximal and distal stumps of the facial nerve several weeks after transection, we treated wild-type mice with F-NP41 intravenously followed by imaging. We found that several weeks following transection, positive identification of the facial nerve stumps were made with more confidence in animals treated with F-NP41 using fluorescent optical microscopy (Fig. 5B) compared to untreated animals under white-light microscopic visualization (Fig. 5A). This is likely due to the strong epineurial staining as shown in Figure 2E and G.

### Operative Time

Measuring the time from skin incision to time of nerve stump identification for both proximal and distal ends, we found that this time was 28 ± 6.1 minutes with the use of F-NP41 and 18 ± 5.8 minutes without (Table I, two-tailed *t*-test, *P* = .001). This longer time to nerve identification in the case of F-NP41 use was due to the added steps of 1) setting up the fluorescence microscopy, 2) adjusting the focal plane for both white-light and fluorescein imaging, and 3) changing back and forth between fluorescent and white-light microscopy to confirm nerve identity in both channels.

**TABLE I:** Summary of Facial Nerve Transection Results

Group	N	Facial Movement (Postop Week 8)	Time to Nerve Recovery (Days)	Operative Time (Min)
Without NP	9	6 (67%)	36 ± 11	18 ± 5.8
With NP	9	8 (89%)	42 ± 25	28 ± 6
	18	*P =* 0.26	*P* = 0.58	*P =* 0.001

N = number; NP = nerve peptide. [Correction added after online publication, February 16, 2011: In Table I, several values have been corrected as some of the animals were excluded from final analysis due to reviewer comment. We apologize for this error.]

### Functional Recovery

The average time for evidence of functional recovery from the date of anastomosis was 36 ± 11 days in mice not treated with F-NP41 versus 42 ± 25 days in mice treated with F-NP41 (Table I, two-tailed *t*-test, *P* = .58). Overall functional recovery for mice whose nerves were reanastomosed (with and without F-NP41) was 78% (14/18) versus 14% (1/7) in the group that did not undergo reanastomosis (Pearson's chi test, *P* = .004). Functional recovery was 67% (6/9) without F-NP41 and 89% (8/9) with F-NP41 (Table I, Pearson's chi test, *P* = .26).

### Complications

There were two perioperative deaths due to overwarming, two from anesthetic overdose, and one from direct arterial anesthetic injection. In the F-NP41-treated group, two cases of ipsilateral corneal ulcer healed without intervention. One mouse in the non F-NP41-treated group developed ipsilateral cataract. This was not statistically significant (*P* = .53).

## DISCUSSION

Nerve labeling previously has depended on retrograde or anterograde tracing of individually identified axonal tracts via the use of fluorescent dyes.^9^–^12^ However, these methods have numerous disadvantages. First, depending on where the dye has been injected, this technique can label only one nerve fiber tract at a time. Second, this technique results in only limited labeling with fluorescent dyes along the axonal tracts, because retrograde axonal tracers typically accumulate in the neural cell body. Third, retrograde transport is relatively slow (on the order of millimeters per day), and therefore takes a long time to label human nerves, which are often longer than a meter, such as in the case of the sciatic nerve and its arborizations. Fourth, the application of fluorescent dyes to innervation targets such as direct intramuscular injections to label motor nerves results in variable amounts of the tracer dye remaining at the injection site, thereby limiting accurate visualization of adjacent anatomic structures. Finally, the direct injection of the fluorescent dye itself may be damaging to the target organs or nerve of interest, either by mechanical damage or by the very high local concentration of dye and vehicle.

In this study, we evaluated the pattern of facial nerve labeling with a novel fluorescently labeled probe. We also tested whether the use of F-NP41 resulted in accurate nerve stump identification after several weeks nerve transection. We found excellent labeling of intact and transected facial nerves following F-NP41 administration. Several weeks following nerve transection, fluorescently labeled NP41 provided accurate identification of the proximal and distal nerve stumps. Following reanastomosis, time to recovery and level of functional recovery was similar in the absence and presence of F-NP41. Although the operative time required to identify the nerve stumps was slightly longer with the use of F-NP41 than without due to the added steps of fluorescence microscopy, this is likely to be experience dependent. We expect that with further engineering and more routine use of the imaging equipment, the time lost by the added steps for fluorescence imaging will be compensated by faster and more accurate nerve identification. Experiments to determine what, if any, is the maximal amount of time after transection that facial nerves can be labeled with F-NP41, are ongoing. We hypothesize that at longer intervals between transection and reexploration, nerve stump identification will be increasingly difficult with white light illumination. In this situation, the use of F-NP41 may actually reduce operative time and improve functional recovery. On the other hand, the question of how long can one wait after nerve transection to perform reanastomosis in a mouse remains unknown. It is possible that our murine model of facial nerve transection does not actually present a situation that is difficult enough to identify the stumps that our fluorescent probe use would make a statistically significant difference. We are actively developing other murine models of nerve injury that would more accurately portray the eventual use of F-NP41 in humans; for example, a thyroid carcinoma model that makes localization of the recurrent laryngeal nerve highly challenging. We are also introducing squamous cell carcinoma into the parotid region that might cause difficult identification of the facial nerve. Finally, we are exploring the use of larger animal models such as rabbits to better emulate the human scenario.

## CONCLUSIONS

We tested the ability of a recently developed, systemically applied, fluorescently labeled nerve probe to aid in the visualization of facial nerves. We found improved visualization of facial nerve branches, especially ones not immediately on the surface of the surgical field using the novel fluorescently labeled probe compared to white-light illumination. We also found that several weeks following transection, facial nerve stumps can still be labeled with the fluorescently labeled probe. There was no apparent toxicity noted with the use of F-NP41. The percentage of animals that recover facial nerve function after transection and reanastomosis, and the time it took for recovery of function were similar between treated and untreated animals. Although new technology can be challenging to implement especially if it requires familiarity with new equipment for detection, we believe that clinical use of this probe for nerve visualization should help surgeons avoid inadvertent transection and repair injured nerves.
